# Microalgae as Potential Anti-Inflammatory Natural Product Against Human Inflammatory Skin Diseases

**DOI:** 10.3389/fphar.2020.01086

**Published:** 2020-07-31

**Authors:** Wu-Thong Choo, Ming-Li Teoh, Siew-Moi Phang, Peter Convey, Wei-Hsum Yap, Bey-Hing Goh, John Beardall

**Affiliations:** ^1^School of Biosciences, Taylor’s University, Lakeside Campus, Subang Jaya, Malaysia; ^2^Institute of Ocean and Earth Sciences, University of Malaya, Kuala Lumpur, Malaysia; ^3^National Antarctic Research Centre, Institute of Graduate Studies, University of Malaya, Kuala Lumpur, Malaysia; ^4^Faculty of Applied Sciences, UCSI University, Kuala Lumpur, Malaysia; ^5^British Antarctic Survey, NERC, Cambridge, United Kingdom; ^6^Biofunctional Molecule Exploratory Research Group (BMEX), School of Pharmacy, Monash University Malaysia, Bandar Sunway, Malaysia; ^7^College of Pharmaceutical Sciences, Zhejiang University, Hangzhou, China; ^8^School of Biological Sciences, Monash University, Clayton, VIC, Australia

**Keywords:** microalgae, bioactive compounds, anti-inflammation, anti-oxidant, skin disease

## Abstract

The skin is the first line of defense against pathogen and other environmental pollutant. The body is constantly exposed to reactive oxygen species (ROS) that stimulates inflammatory process in the skin. Many studies have linked ROS to various inflammatory skin diseases. Patients with skin diseases face various challenges with inefficient and inappropriate treatment in managing skin diseases. Overproduction of ROS in the body will result in oxidative stress which will lead to various cellular damage and alter normal cell function. Multiple signaling pathways are seen to have significant effects during ROS-mediated oxidative stress. In this review, microalgae have been selected as a source of natural-derived antioxidant to combat inflammatory skin diseases that are prominent in today’s society. Several studies have demonstrated that bioactive compounds isolated from microalgae have anti-inflammation and anti-oxidative properties that can help remedy various skin diseases. These compounds are able to inhibit production of pro-inflammatory cytokines and reduce the expression of inflammatory genes. Bioactive compounds from microalgae work in action by altering enzyme activities, regulating cellular activities, targeting major signaling pathways related to inflammation.

## Introduction

The skin is the outermost layer and considered the largest organ in the human body. It plays an important protective role by providing a major boundary between the host and the external environment (Benson and Watkinson, 2012). The skin is also well equipped with effective defenses against pathogens and other environmental pollution (Wang H. et al., 2013). Exogenous threats such as UV radiation and oral introduction of potentially toxic dietary and drug metabolites, all of these factors may influence the health and appearance of the skin (Sander et al., 2004). The body is constantly exposed to these environmental agents and endogenous metabolites that may have either short-term or long-term side effects to the host. Because of that, they may directly or indirectly promote the production of reactive oxygen species (ROS) that stimulate the inflammatory process in the skin (Kohen, 1999; Trouba et al., 2002). The surface of the skin is constantly exposed to ROS as its first line of defense against pathogens and external pollutants. Many studies have linked ROS to various inflammatory skin diseases (atopic dermatitis [AD], psoriasis, and vitiligo), skin aging, and carcinogenesis (Briganti and Picardo, 2003; Sander et al., 2004; Okayama, 2005).

Skin diseases are the fourth most common cause of non-fatal disease burden and 18^th^ leading cause of global disability-adjusted life years (DALYs) worldwide within the year of 2010–2013 (Karimkhani et al., 2017). Dermatitis (consisting of atopic, seborrheic, and contact categories) is the highest burden among of the skin conditions, make up a total of 9.3 million DALYs (Karimkhani et al., 2017). Skin diseases may present similarly across racial and ethnic groups; however, some features may be either more prominent in patients with darker skin (Mei-Yen Yong and Tay, 2017; Kaufman et al., 2018). Besides that, patients with skin diseases face challenges with ineffective and inappropriate treatment, such as oral antihistamines, oral corticosteroids, or traditional medicines, which may have low potency or have significant side effects for certain individual (Lopez Carrera et al., 2019). Therefore, natural derived ingredients may be potential in combating against skin diseases (Jahan et al., 2017).

ROS consist of reactive molecules and free radicals that are oxygen based and are often associated with the principle of oxidative stress, which suggests that ROS induce cell damage by interfering with lipids, proteins, and DNA within the body (Cross, 1987). ROS are produced during the reduction of molecular oxygen as follow:

$$O_{2}+1e^{-}+H^{+}\to H_{2}O\to H^{+}+O_{2}^{-}$$

$$H_{2}O+1e^{-}+H^{+}\to H_{2}O_{2}$$

$$H_{2}O_{2}+1e^{-}+H^{+}\to H_{3}O_{2}\to H_{2}O+OH$$

$$OH+1e^{-}+H^{+}\to H_{2}O$$

Superoxide anion (O_2_^-^), hydroxyl radicals (OH.), hydrogen peroxide (H_2_O_2_), and molecular oxygen (O_2_) at low levels in the body are involved in various cellular process such as, cell proliferation, apoptosis, immune responses, and cell differentiation (Kumar and Pandey, 2015). In contrast, overproduction of ROS will result in oxidative stress, which will further lead to altered metabolism, dysregulated signal transduction, and biomolecular cell damage, which cause pathological changes in normal cell function (Trouba et al., 2002). Biomolecular damage that occurs as a result of increasing ROS levels has considered as lipid peroxidation, DNA mutation, enzyme inactivation/activation, and protein oxidation/degradation. Such damages will usually cause further damaging effects as the result of ROS. Thus, the amount of ROS level is displayed to be hormesis, whereby low dose stimulation showing beneficial effects and high dose stimulation showing toxic effect (Di Meo et al., 2016).

## Relationship Between Oxidative Stress and Skin Inflammation Disease

**Figure 1:**
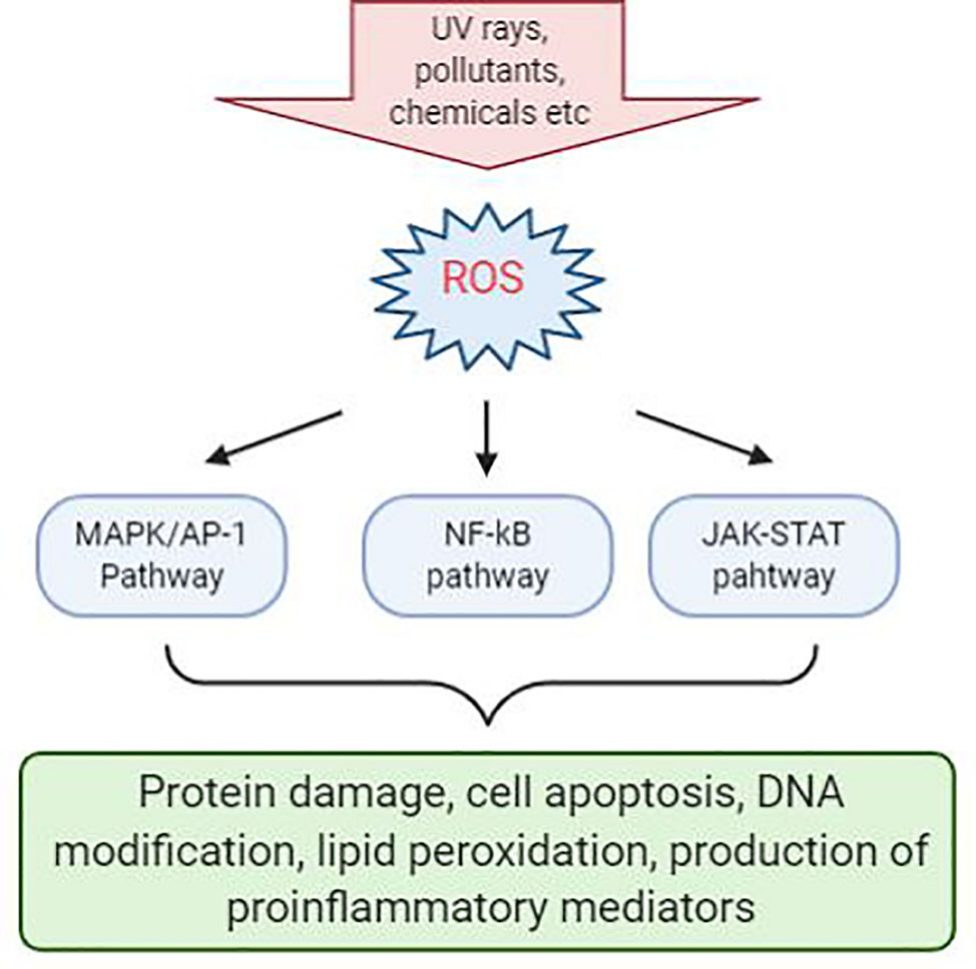
Reactive oxygen species (ROS)-mediated activation of various cell signaling pathways in the skin. Chemical irritants, allergens, or inflammatory agents that cause the production of ROS during the pathogenesis of various skin disease and activation of a various number of signaling pathways. Signaling pathways with the most significant effects are MAPK/AP-1, NF-κβ, and JAK-STAT pathways. Activation of these pathways will result in protein damge, cell apoptosis, DNA modification, lipid peroxidation, and production of proinflammatory mediators.

Inflammation triggers when the body detects the presence of pathogens or irritants that are present in the body. Many studies have linked that there is correlation between oxidative stress and various inflammatory skin disease. Evidence shows that ROS-mediated oxidative stress stimulates the production of oxidative products which can cause damage to proteins, triggers cell apoptosis, cause DNA modification, lipid peroxidation, and promotes the release of proinflammatory mediators, such as cytokines and chemokines, which may be the main cause of several inflammatory skin disease to occur (Meffert et al., 1976). In addition, ROS acts as a secondary messenger in influencing cellular signal transduction pathways proinflammatory signaling pathways and modulate the expression of several gene involved in inflammation (Finkel and Holbrook, 2000). The most significant effects were seen in MAPK/AP-1, NF-κβ, and JAK-STAT signaling pathways during early stage of every inflammatory disorders (**Figure 1**) (Dhar et al., 2002; Wolk et al., 2004; Kim et al., 2005; Wang et al., 2006). Activation of these signal transduction cascades results in the production of growth factors, cytokines, neurotransmitters, and other signaling molecules, thus leading to cell proliferation, differentiation, and apoptosis.

## Mechanism of Anti-Oxidants in Skin

Anti-oxidants functions to delay or prevent ROS-induced cellular damage. They can help to reduce oxygen radicals, inhibiting chain initiation reactions, binding catalysts that generate ROS, and attenuating hydrogen radicals (Trouba et al., 2002). Anti-oxidants can be categorized as enzymatic and non-enzymatic in the intracellular and extracellular environment (Frei et al., 1988).

Rice-Evans and Diplock (1993) proposed that anti-oxidants had two principle mechanism of action against ROS. The first is a chain breaking mechanism whereby anti-oxidant donates an electron to the free radical that is present in the systems. The second mechanism involves removal of ROS/RNS initiators by quenching chain-initiating catalyst. Anti-oxidants may cultivate different mechanism to combat radicals in the biological systems such as, electron donation, metal ion chelation, co-antioxidants, or by gene expression regulation (Krinsky, 1992).

## Skin Disorders

### Atopic Dermatitis

**Figure 2:**
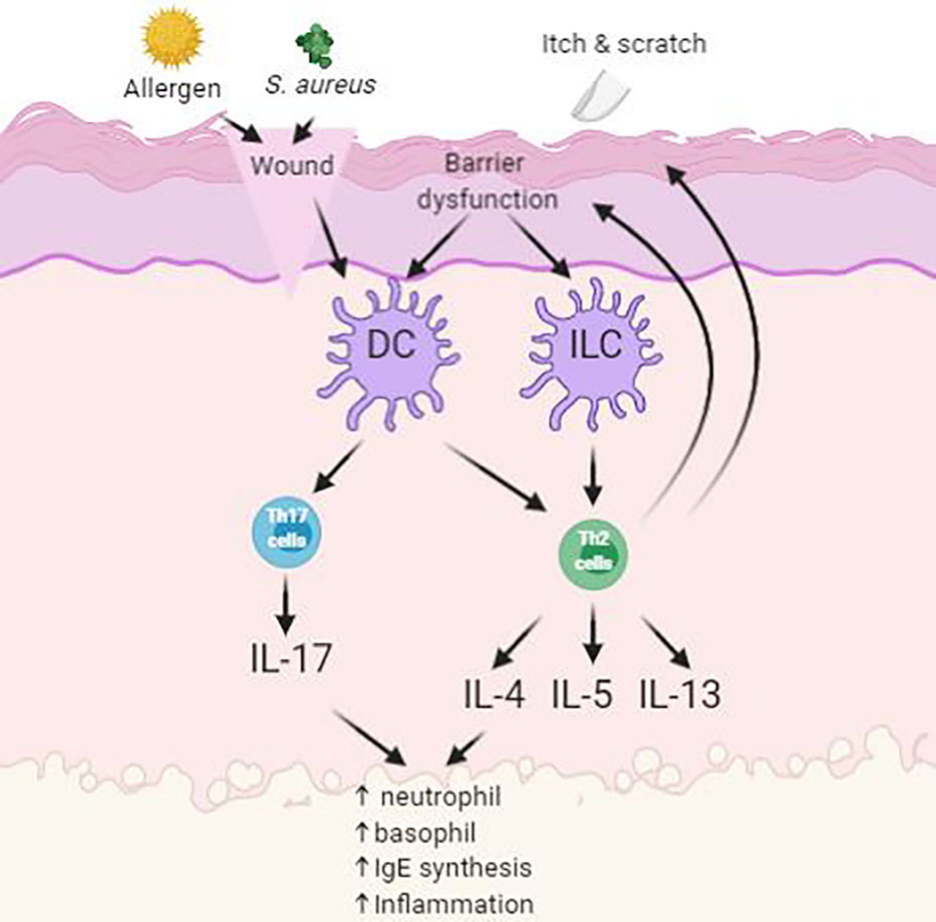
Simplified schematic of pathogenesis of atopic dermatitis (AD) with skin barrier defect. Impact of infections, allergens and itch leads to upregulation of inflammatory pathways.

Atopic dermatitis (AD) is a chronic, itching, inflammatory disease and is predominantly between 10 and 20% of pediatric population (Kowalska-Olędzka et al., 2019). Symptoms of AD appear in 60% of patients before reaching 1 year of age (Kowalska-Olędzka et al., 2019). AD is believed to be caused by combination of genetic and environmental factors (Eigenmann and Calza, 2000; Hill and Hosking, 2004). It is characterized by having papules and plaques. The earliest lesion is a small erythematous papule or papulovesicle. These papules may then later become erythematous plaques with clinical features of weeping, crusting, or scaling, depending on the severity of the lesions form (Simpson, 2010). The most problematic symptom of AD is itch. The “itch-scratch” cycle involves scratching on affected areas on the skin to relieve AD associated itch and would further worsen the disease (**Figure 2**). These symptoms would usually accompany with sleep disturbance and social stigma of visible skin disorder, therefore having negative impact to the quality of life to the patient and family (Bender et al., 2008).

Symptoms of AD often reduces the barrier function of the skin as demonstrated by elevated trans-epidermal water loss and increase permeation of environmental irritants and allergens. Antigen-presenting cells (APC), such as Langerhan’s cells, process antigens and present it to lymphocytes, where the process will stimulate helper T-cells into the skin. These cells then stimulate the production of proinflammatory cytokines into the inflamed area. This will then enable the activation of imbalanced growth factors, cytokines (IL-1, IL-6, GM-CSF, TNF-α), and chemokines that interfere with the normal mechanism of the innate immune system of the skin (Nesterova et al., 2019). Overproduction levels of cytokines will oppose the mechanism of innate immunity in the skin and consequently cause secondary bacterial infection at site of inflammation (Albanesi and Pastore, 2010). Patients with AD are prone to acquiring secondary cutaneous infections such as *Staphylococcus aureus* infections. These infections are usually present in the form of impetigo or folliculitis due to further worsening from scratching (Leyden et al., 1974).

Other than avoiding irritants and moisturizing the skin with emollients, the most common method to treat AD is by applying topical corticosteroids (Spergel, 2008). Topical corticosteroids produce instant results in short-term treatment on skin itching and inflammation of skin. However, long-term usage of such treatment may lead to adverse effects, such as skin atrophy and telangiectasia (Van der Aa et al., 2010). Besides that, long term use of topical corticosteroids will cause thinning of the stratum corneum, therefore allowing irritants and allergens entry into the skin.

### Psoriasis

Psoriasis is a chronic autoimmune disease characterized by raised, red scaly plaques (Lowes et al., 2014). This disease affects 2–3% of the world population, resulting in psychological stress and poor quality of life (Perera et al., 2012). The most common type of psoriasis is psoriasis vulgaris which accounted about 85–90% of all cases of psoriasis. Psoriasis is caused by hyperproliferation of keratinocytes and infiltration of activated immune cells triggered by several factors such as physical and psychological stress, bacterial infections, or injury (Dika et al., 2007; Lowes et al., 2007).

**Figure 3:**
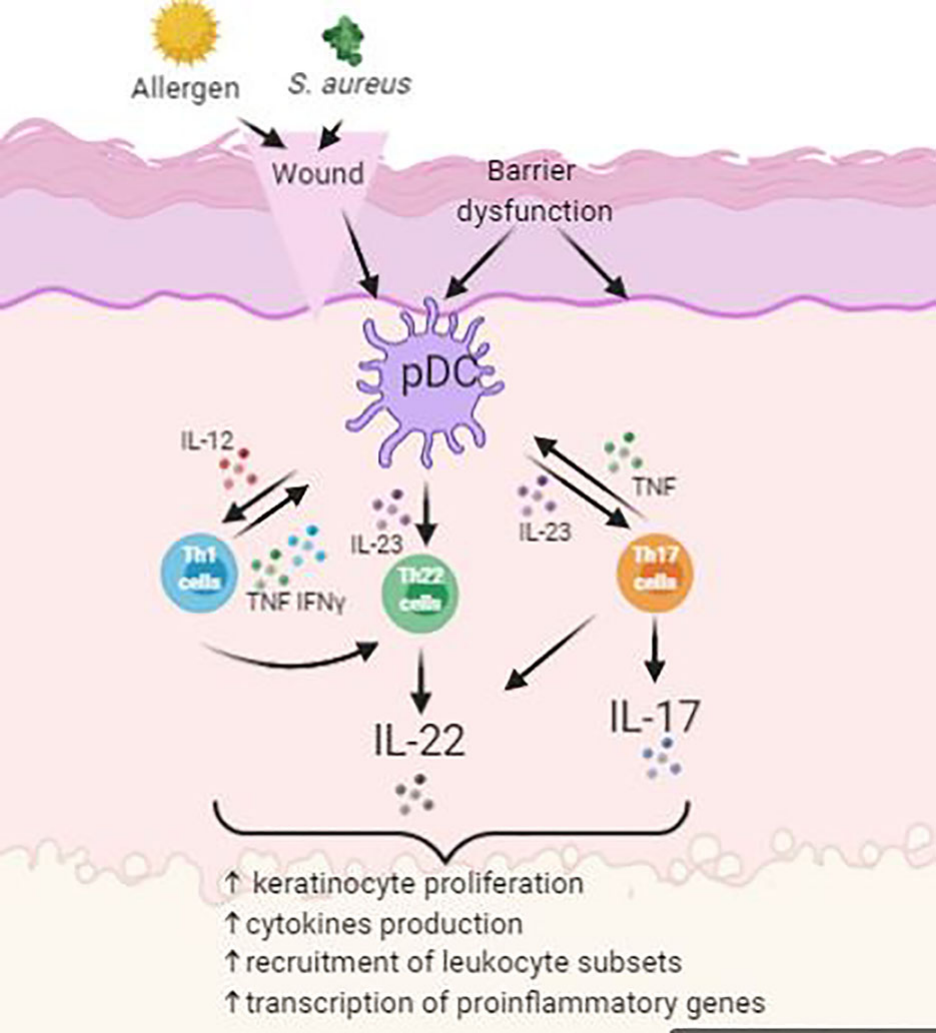
Simplified schematic of pathogenesis of psoriasis with activation of Th22 and Th17 cells.

Several studies have identified that overexpression of various cytokines occurred in psoriasis, such as interleukins (ILs), tumor necrosis factor (TNF), and interferon-γ (IFN-γ) (**Figure 3**) (Nickoloff et al., 2007). Furthermore, development of psoriasis was closely linked to complex cellular interactions among epidermal keratinocytes, leukocytes, neutrophils, dendritic cells, and activated T cells, growth factors, cytokines, and chemokines (Nickoloff et al., 2007).

The mitogen-activated protein kinase signaling pathways composed of extracellular-regulated kinase (ERK1/2 and ERK3/4) (Seternes et al., 2004), Jun N-terminal kinase (JNK) (Davis, 1994; Cano and Mahadevan, 1995), p38 kinase (Cano and Mahadevan, 1995; Cobb and Goldsmith, 1995), and big mitogen activated protein kinase 1 (BMK1) (Kato et al., 2000). Several studies have demonstrated that ROS may trigger pathogenesis of psoriasis through ERK1/2, JNK, and p38 MAPK pathways (Takahashi et al., 2002; Johansen et al., 2005; Yu et al., 2007). However, no reports have found that ERK3/4 and BMK1 pathway being involved in the pathogenesis of psoriasis due to oxidative stress as of now.

During cell stimulation, Iκβ (inhibitor of κβ) proteins are rapidly phosphorylated and degraded by the proteasome, the unbound NF-κβ translocate into the nucleus to regulate multiple gene expressions (Hayden, 2004). These genes may encode for TNF super family, IL-1, IL-6, IL-8, iNOS, major histocompatibility complex class 1 (MHC class 1) antigens, E-selectin, and vascular cell adhesion molecule 1 (VCAM-1), which are mostly involved in psoriasis (Wajant et al., 2003; Schottelius et al., 2004).

Although MAPK/AP-1 and NF-κβ signaling pathways is triggered by ROS in the pathogenesis of psoriasis, there is solid evidence showing that there’s a correlation between these two signal transduction pathways. This is because the response to AP-1 is highly stimulated due to the presence of NF-κβ and thus NF-κβ positively modulates the expression of c-Fos and AP-1 activity (Stein et al., 1993; Fujioka et al., 2004).

The JAK-STAT signal pathway also plays an important role in immune and inflammatory responses (Kishimoto et al., 1994). Studies shown that STAT1 is upregulated by IFN-γ and IL-20 and thus induce inflammatory mediators in a type 1 cytokine pathway model of psoriasis pathogenesis (Wang et al., 2006). Another study had demonstrated that STAT3 pathway is believed to play a role in the pathogenesis of psoriasis where STAT3 is activated by IL-22 and result in the increased expression of β-defensin 2/3 (Wolk et al., 2004).

The most common conventional treatment of psoriasis consists of corticosteroids, Vitamin D analogues, phototherapy, and systemic treatments (Novelli et al., 2014). However, long-term usage of conventional treatments may cause severe health issues to patients, such as poor tolerability and cumulative toxicity (Rahman et al., 2012). Topical glucocorticosteroids and Vitamin D analogues are commonly used to treat psoriasis by regulating keratinocyte function and inflammatory response. Nonetheless, long term treatment of corticosteroid can cause several health issues, such as cutaneous atrophy, short remission duration, and cause psoriasis to be rebound (Chong and Fonacier, 2015). Regardless of their reduce side effects in comparison to corticosteroid, vitamin D analogues still fail to have a rapid activation in the body (Smith and Barker, 2006). Another widely used treatment for psoriasis, methotrexate, due to its reasonable price and its high efficacy (Boehncke, 2003; Heydendael et al., 2003). However, prolonged usage of the drug may cause patients to suffer liver fibrosis and cirrhosis (Deng et al., 2016).

### Vitiligo

Vitiligo is a skin disorder characterized by depigmented macules from melanocyte dysfunction in the epidermis (Boniface et al., 2017). Vitiligo affects approximately 0.5–1% of the world population and both genders are equally affected (Taïeb and Picardo, 2009). Studies have suggest that destruction of melanocytes is the main cause of the pathogenesis of vitiligo (Poole et al., 1993). Oxidative stress may be the main pathogenic reason in melanocyte loss (Maresca et al., 1997; Schallreuter, 1999).

Patients in active phase of vitiligo has high levels of ROS in the epidermal layer, primarily consists of hydrogen peroxide (H_2_O_2_) and peroxynitrite (Schallreuter et al., 1991; Schallreuter et al., 1999). This damage is due to local and systemic imbalance in enzymatic and non-enzymatic anti-oxidant systems in the skin (Shajil and Begum, 2006). Studies have demonstrated that vitiligo patients have high levels of tetrahydrobiopterin (6BG4) and the isomer 7BH4 in the epidermis and inhibits the enzymes involved in melanogenesis and increases the production of H_2_O_2_ (Schallereuter et al., 1994; Hasse et al., 2004).

In melanocytes, the alteration of calcium homeostasis due to the increase in level of H_2_O_2_ systemically and locally would indirectly interfere with the intake of L-phemylalanine, an amino acid precursor of tyrosine (Schallreuter et al., 2007). Proopiomelanocortin-derived bioactive peptides ACTH and a-MSH plays crucial role in melanogenesis by activating a cascade of intracellular signals that promotes relevant enzymes in melanin production, such as tyrosinase and tyrosinase-related proteins 1 and tyrosinase-related proteins 2. Presence of ROS may oxidize or inhibit these signaling pathways for melanin synthesis (Tachibana, 2000).

Accumulation of ROS may induce DNA damage, lipid peroxidation, increased production of proinflammatory, and antimelanogenic cytokines (Mahendra et al., 2019a; Mahendra et al., 2019b). Altered enzymes that play a key role in melanogenesis may show partial or complete dysfunction due to damage done from oxidative stress (Denat et al., 2014).

Recent studies have linked Nrf2-anti-oxidant response element (ARE) pathway being affected by oxidative stress in regulating vitiligo skin homeostasis (Jian et al., 2011; Jian et al., 2014; Qiu et al., 2014). Nrf2-ARE is an anti-oxidant pathway that regulates the transcription of stress-related cytoprotective genes and thus protecting cells from radical molecules (Jian et al., 2011). One example of such gene is the heme oxygenase-1 (HO-1). Another study had shown that vitiligo melanocytes have decrease HO-1 expression and abnormal redox balance due to reduced Nrf2 nuclear translocation and transcriptional activity. For this reason, the same studies were conducted in a clinical setting and showed that vitiligo patients have low expression levels of HO-1 gene when compared to healthy controls (Jian et al., 2014).

Corticosteroids are still the first line therapy for vitiligo and can use either as topical or systemic treatment. Topical corticosteroids help to decrease the destruction of melanocyte and repopulate melanocyte and melanin production (Bleehen, 1976; Hann et al., 1993). However, corticosteroids still pose to have various side effects which includes skin atrophy, telangiectasia, and striae distensae, steroid folliculitis, and acne formation on the skin (Speeckaert and van Geel, 2017). In contrast, oral corticosteroid therapy in moderate doses helps in arresting the progression of vitiligo (Speeckaert and van Geel, 2017). Side effects include acne formation, disturbance in sleep patterns, weight gain, agitation, hypertrichosis, and menstrual abnormalities, when consume on a long-term basis (Radakovic-Fijan et al., 2001).

Phototherapy is also a common treatment to induce repigmentation in vitiligo patients. Phototherapy involves usage of both UVA and UVB to promote melanocyte migration and proliferation (Wu et al., 2007). Common side effects patients may receive from psoralen and ultraviolet a (PUVA) treatment includes erythema, pruritus, headache, and nausea (Valkova et al., 2004). Patients may also suffer from second-degree burns when incorrect radiation doses from phototherapy (Herr et al., 2007). PUVA also carries a higher risk in gaining non-melanoma skin cancer and melanoma (Park et al., 2003; Patel et al., 2009).

## Microalgae as Natural Source of Bioactive Compounds

Microalgae are unicellular microorganism that can be found in freshwater or marine environments. They are autotrophic as they are capable to undergo photosynthesis with the aid of sunlight, they can convert water and carbon dioxide into organic compounds like terrestrial plants (Metting, 1996). Microalgae have many advantages over terrestrial plants as they are fast growing, easily cultivate, and do not compete directly with agricultural crops. Microalgae are capable to live in extreme conditions and environment by adopting surviving strategies by producing various bioactive compounds with diverse structure and unique activity to counter environmental stress (Christaki et al., 2012). Therefore, microalgae has drawn great attention as candidate for natural products to be use in the medical, pharmaceutical, cosmetic, and biofuel industries (Sathasivam et al., 2019).

Microalgae produce various bioactive compounds that exhibit potential pharmacological effects, including anticancer, antidiabetic, anti-inflammatory, and anti-oxidative activities (Fu et al., 2017). These bioactive compounds include fatty acids, phycobiliproteins, chlorophylls, carotenoids, and vitamins. Microalgae-derived bioactive compounds have been proven to be able to overcome inflammatory skin disorders, given by their tremendous structural diversity and biological availability. Furthermore, the culture condition of microalgae can be easily manipulate to favor production of specific bioactive compounds by addition or removal of certain nutrients (Fu et al., 2017).

Bioactive compounds of microalgae from primary metabolism are directly involved in cell growth and reproduction. Primary metabolites from microalgae are usually consists of carbohydrates, lipid, and proteins (Wen et al., 2015). Secondary metabolites have gained several attentions over the past decades due to their wide health benefiting properties. These metabolites are mainly involved in adaption of microalgae to the environment (Bourgaud et al., 2001). In most microalgae species, the bioactive compounds are accumulated in the form of their biomass; while in some cases, they are excreted into the medium.

### Protective Effects of Microalgae Against Inflammatory Skin Diseases

Microalgae extracts possess anti-inflammatory properties as they are able to inhibit production of pro-inflammatory cytokines and reduce the expression of inflammatory genes (Robertson et al., 2015). Bioactive compounds from microalgae works in various ways in inhibiting skin inflammation which includes altering enzyme activities, regulating cellular activities, nitric oxide synthase (NOS), and targeting major signaling pathways, such as NF-ƙβ and MAPKs pathway (Hussain et al., 2016). Both of these pathways are major mediators of various inflammatory producer. Besides having anti-inflammatory, microalgae extracts may also possess anti-oxidative properties too (Arulselvan et al., 2016).

## Key Metabolites in Microalgae

### Carotenoids

Carotenoids are mostly found in plants, marine algae, fungi, and bacteria. Carotenoids are divided into two classes, carotenes and xanthophylls (Sathasivam and Ki, 2018). Xanthophylls contain oxygen group in their structure while it is absent in carotenes. Carotenoids have a common chemical backbone which composed mainly of polyene chain with a long conjugated double bond system. The ending of the chain may be terminated with cyclic groups containing oxygen-bearing substitutes. The electron-rich conjugated system of the polyene and its cyclic end groups determine the functional anti-oxidant properties of carotenoids (Martin et al., 1999).

In microalgae, carotenoids play a role in protecting the chlorophyll from long-term exposure of light by scavenging ROS, phototropism, and photoaxis (Esteban et al., 2009). Carotenoids can scavenge harmful radicals through three different ways, which are, electron transfer, radical adduct formation, and hydrogen atom transfer (Burton and Ingold, 1984; Rice-Evans et al., 1997; Paiva and Russell, 1999; Skibsted, 2012).

$$Car+R^{•}\to Car^{•+}+R^{-}\;(electron\;transfer)$$

$$Car+R^{•}\to [Car\cdots R{]}^{•}\;(radical\;adduct\;formation)$$

$$Car+R^{•}\to Car(-H{)}^{•}+RH\;(hydrogen\;atom\;transfer)$$

Carotenoids are potential to be used to treat and control chronic inflammation as research shown that they are capable to inhibit pro-inflammatory cytokines (Zhang et al., 2014). Carotenoids are able to regulate chronic inflammatory disorder by inhibiting the effects from nitric oxide production, pro-inflammatory cytokines, expression of pro-inflammatory genes, and pro-inflammatory enzyme activities (Hussain et al., 2016).

#### Astaxanthin

Astaxanthin is a lipid soluble pigment and is classified as a xanthophyll. It is a secondary metabolite and is mainly found in the marine environment as a red-orange pigment. Astaxanthin is primarily synthesized naturally by microalgae, zooplankton, crustaceans, and certain species of fish, such as salmonids. Although astaxanthin can be synthesized from plants, bacteria, and microalgae, one particular species of microalgae, namely *Haematococcus pluvialis*, is known to produce the highest content of astaxanthin (Boussiba, 2000).

Astaxanthin has reported to have higher bioactive properties than zeaxanthin, lutein, and β-carotene. This is due to the presence of a keto- and hydroxyl group on each end of its structure. Because of its unique structure, astaxanthin has the potential to be used in promoting human health. The polar end groups of astaxanthin are able to quench free radicals, the double bond chains of the structure help remove high-energy electrons. Because of its unique structure, astaxanthin has a higher anti-oxidant activity than other carotenoids (Higuera-Ciapara et al., 2006). When it come to the characteristics of its existence, astaxanthin is polar in nature and hence its rate in absorption is rather easy with a moderate consumption. Besides the integrity of membrane is preserved by conveniently instilling themselves in between bilayers. With this feature, astaxanthin can also preserve the functionality of the mitochondria that lead to the protection of the redox state of the body (Wolf et al., 2010; Kidd, 2011).

Studies on rat models on the effects of astaxanthin on lipopolysaccharide-induced inflammatory reactions shows that astaxanthin (100 mg/kg) has a higher anti-inflammatory activity than that of 10 mg/kg of prednisolone, a common anti-inflammatory drug. Further results showed that astaxanthin inhibits production of NO, prostaglandin E_2_ (PGE_2_), TNF-α, and interleukin-1β, and also blocks the NOS enzyme in RAW 264.7 cells (Ohgami et al., 2003).

Other reports stating that human neutrophil treated with 5 mM astaxanthin has improved phagocytic and microbicidal activity. Besides that, oxidative damage to proteins and lipids in human neutrophil were significantly lower after astaxanthin treatment due to the fact that astaxanthin is effective in quenching ROS (Macedo et al., 2010).

Another study has demonstrated that U937 cells (human lymphoma cell line) pre-incubated in 10 mM astaxanthin before inducing H_2_O_2_ showed lower cytokines levels than cells that were not treated with astaxanthin. In addition, cells that were pre-incubated in astaxanthin had shown higher levels of SHP-1 (protein tyrosine phosphate), an enzyme that removes phosphate groups from phosphorylated tyrosine residue from proteins, and lower levels of NF-ƙβ expression (Speranza et al., 2012).

Studies also shown that HaCaT keratinocytes treated with astaxanthin were able to decrease UV-induced release of migration inhibitory factor (MIF), 1L-1β, and TNF-α at both the protein and mRNA levels. Astaxanthin treatment were able to reduce the UVB-induced production of pro-inflammatory cytokines, thus inhibited the cells from undergoing apoptosis and also protected the skin from inflammation. Furthermore, astaxanthin treatment significantly reduced the UVB-induced caspase-3 and caspase-9 activity while no reduction was observed in UVC-induced caspase-3 activity. Therefore, astaxanthin treatment may have a stronger protective effect against UVB-induced apoptosis than UVC-induced apoptosis. Besides that, astaxanthin also decreases the expression levels of inducible nitric oxide synthase (iNOS) and cycloosygenase-2 (COX-2) and decreases production of PGE_2_ in UV-induced HaCaT keratinocyte (Yoshihisa et al., 2014).

#### Lutein

Lutein is a yellow pigment and is classified as a xanthophyll. Lutein helps protect green microalgae from ROS damage. Microalgae have been considered the main natural source for lutein production as they produce higher lutein content compare to plants source (Lin et al., 2015). *Dunaliella salina* has been shown to be potential in producing lutein by manipulating its growth conditions (Fu et al., 2012). Other microalgae strain, such as *Chlorella sorokiniana* and *Chlorella prothecoides* have also been suggested as source of lutein production by manipulating growth conditions (Shi et al., 2002; Cordero et al., 2011). Lutein has been widely studied in the treatment of macular degeneration. However, studies suggest that lutein is capable of quenching oxygen radicals and scavenging free radicals (Jahns and Holzwarth, 2012).

### Microsporine-Like Amino Acid

Studies have proven that mycosporine-Gly possess anti-oxidative activity and may protect against skin inflammation caused by UV radiation. Anti-oxidative activity of mycosporine-Gly (0.3mM) extracted from *Chlamydomonas hedleyi* was high and UV-elevated COX-2 gene were suppressed. This shown that the regulation of COX-2 may be link to the oxidative process from mycrosporine-Gly (Suh et al., 2014).

### Sterols

Sterols are found in almost all living organism. Microalgae produce a wide variety of sterols and plant derivative sterols are termed as phytosterols. Phytosterols extracted from microalgae found to possess anti-inflammatory properties (Yasukawa et al., 1996). A study demonstrated that extract from the microalgae, *Nannochloropsis oculata*, was able to induce anti-inflammatory effect on RAW 264.7 cells. Anti-inflammatory of the extract were accomplished by the decrease expression of iNOS and COX-2 proteins (Sanjeewa et al., 2016).

### Polysaccharides

The cell walls of microalgae are rich in sulphated polysaccharide (SPs) and it exhibit various health benefiting properties such as anticoagulant, anti-oxidant, antiviral, anticancer, and anti-inflammatory activities (Wang J. et al., 2013; De Jesus Raposo et al., 2015). Therefore, SPs from microalgae have potential to be incorporate in nutraceutical, pharmaceutical, and cosmeceutical products. The biological and pharmacological activities of SPs are due to the complex interaction of the structure feature, such as the sulphation level, distribution of sulphate groups along the backbone of the molecule, molecular weight, sugar residue composition, and stereochemistry (Damonte et al., 2004; Ghosh et al., 2009). Several studies have demonstrated SPs for its potent anti-inflammatory properties. For instance, a study on *Chlorella stigmatophora* and *Phaeodactylum tricornutum* had demonstrated anti-inflammatory activity on paw edema. Carrageenan extracted from both microalgae strain had better anti-inflammatory activity on *in vivo* and *in vitro* models compared to the anti-inflammatory drug, indomethacin (Guzmán et al., 2003). Besides that, inhibition of leukocyte migration has a correlation with the anti-inflammatory activity of polysaccharides. Leukocyte movement to site of injury promotes cytokine release and production of nitric oxide. SPs from *Porphyridium* had demonstrated to inhibit the movement and adhesion of polymorphonuclear leukocytes and development of erythema *in vivo* (Matsui et al., 2003).

Besides inhibiting inflammatory cytokines and chemokines, studies also shown that *P. cruentum* is capable of inhibiting biomembrane peroxidation and for its immunomodulatory activities (Sun et al., 2009; Sun et al., 2012). Studies had demonstrated that low molecular fractions of exopolysachharide (EPS) from *P. creuntum* can stimulate the production of macrophages and nitric oxide (NO). NO is a free radical produced by phagocytes and plays a role in the immune system. In addition, other study also justified that SPs from cyanobacteria can promote immune system by triggering the cell and humor stimulation (Namikoshi and Rinehart, 1996).

### Conclusion

ROS are prevalent in nature and are constantly produced at minute amount in aerobic systems. Living organism are capable to produce a wide range of anti-oxidant to eliminate or inhibit ROS to maintain homeostasis. Our body is constantly exposed to various pro-oxidants from the environment, such as consumption of drugs, solar radiation, pollutant, food additives, synthetic cosmetic products, which are capable to induce ROS in skin. ROS mostly target DNA, proteins, and lipid-rich membranes to induce toxicity effects to the body. Such damages to the skin may result in numerous skin diseases ranging from AD to vitiligo. Therefore, discovery of novel anti-inflammatory drugs from natural products that could potentially eliminate or inhibit ROS could bring new insight for biomedical research and industry (Fernando et al., 2016). Microalgae has become the main interest among consumers and industry due to their ability to produce a wide variety of bioactive compounds with health promoting properties and anti-inflammatory can be isolated from them. However, further pre-clinical investigation is still needed to understand the mechanism of these novel compounds in tackling skin inflammation diseases.

## Author Contributions

W-TC, M-LT, S-MP, PC, W-HY, B-HG and JB contributed to the idea and wrote the manuscript. All authors contributed to the article and approved the submitted version.

## Conflict of Interest

The authors declare that the research was conducted in the absence of any commercial or financial relationships that could be construed as a potential conflict of interest.
